# Immunohistochemical Assessment of Microvessel Density in OSCC: Spatial Heterogeneity of Angiogenesis and Its Impact on Survival

**DOI:** 10.3390/biomedicines11102724

**Published:** 2023-10-08

**Authors:** Andreas Mamilos, Alexander Lein, Lina Winter, Markus Haas, Torsten E. Reichert, Tobias Ettl, Julian Künzel, Gerrit Spanier, Christoph Brochhausen

**Affiliations:** 1 Institute of Pathology, University of Regensburg, 93053 Regensburg, Germany; 2 Central Biobank Regensburg, University and University Hospital Regensburg, 93053 Regensburg, Germany; 3 Department of Pathology, German Oncology Center, 4108 Limassol, Cyprus; 4 Department of Otorhinolaryngology, Head and Neck Surgery, Medical University of Vienna, 1090 Vienna, Austria; 5 Institute of Pathology, Medical Faculty Mannheim, Heidelberg University, 68167 Mannheim, Germany; 6 Department of Oral and Maxillofacial Surgery, University Hospital Regensburg, 93053 Regensburg, Germany; 7 Department of Otorhinolaryngology, University Hospital Regensburg, 93053 Regensburg, Germany

**Keywords:** angiogenesis, oral squamous cell carcinoma, microvessel density, spatial heterogeneity, survival, immunohistochemistry

## Abstract

(1) Background Oral squamous cell carcinomas (OSCC) are a common malignancy of the oral cavity and are often diagnosed when they have already spread to the regional lymph nodes. Advanced stages of cancer are characterized by the development of distant metastases. Angiogenesis, a hallmark of cancer, is known to contribute to cancer progression and metastasis. High microvessel density (MVD) has been linked to poor clinical outcomes in various types of cancer. (2) Methods: In this study, we aimed to investigate the spatial heterogeneity of blood vessels by comparing the tumor center and invasion front and to evaluate its prognostic value in OSCC. A total of 71 OSCC patient specimens were collected. The tissue was immunohistochemically stained using CD31 antibody to assess the MVD in the tumor center and the invasion front. Furthermore, the associations between the histopathological parameters, including MVD, disease-free survival (DFS), and overall survival (OS) were computed. (3) Results: In our study, we found a significantly higher presence of blood vessels at the invasion front of OSCCs compared to the tumor center. However, we did not observe any significant differences in MVD between different tumor stages. High intratumoral MVD was shown to be a positive prognostic factor for DFS (*p* = 0.047). (4) Conclusions: To the best of our knowledge, we were the first to analyze MVD as a prognostic factor by considering its spatial heterogeneity in OSCC. However, further studies are warranted to further elucidate the complexity of microvascular spatial heterogeneity and its influence on prognosis.

## 1. Introduction

Oral squamous cell carcinoma (OSCC) represents the most prevalent cancer affecting the oral cavity and ranks as the 18th most common malignancy overall [1,2]. Although significant progress has been made in diagnosis and therapeutic interventions, OSCC continues to have an unfavorable prognosis, depending on patient age, anatomical location, tumor stage, and pathological classification [1]. Due to its aggressive nature, cervical lymph node metastases can be detected in the advanced stages of the disease in as many as 40% of the patients with the first cancer diagnosis [3]. Advanced OSCC is associated with a high risk of developing distant metastases, which are known to have a poor prognosis, with a median overall survival of less than one year [4]. 

Several pathomechanisms are known to be mandatory for the development of distant metastases. Angiogenesis is the formation of new blood vessels from preexisting ones and is considered a hallmark of cancer [5]. Tumor cells and other cells of the tumor microenvironment (TME) secrete angiogenic factors that stimulate the growth of new blood vessels, which allow the tumor to reach a clinically apparent size and provide a pathway for tumor cells to metastasize [6,7]. The morphology of these newly formed blood vessels in cancer is highly heterogeneous. They are often leaky and compressed and display irregular branching patterns, leading to fluctuating blood flow and nutrient delivery to the tumor [8].

Targeting angiogenesis has shown promising results in the treatment of various cancer entities. Anti-angiogenic agents such as bevacizumab and pazopanib aim to inhibit the formation of new blood vessels, thereby aiming at inhibiting tumor growth and metastasis and improving survival outcomes [9]. However, the clinical evidence supporting the use of anti-angiogenic therapies (AAT) in OSCC is currently limited, and no anti-angiogenic drugs have been approved by the FDA for this indication so far [10,11]. In this context, tumors with a higher microvessel density (MVD) appear to have an earlier progression than tumors with a low MVD [12]. Elevated levels of angiogenic factors, such as vascular endothelial growth factor (VEGF) and angiogenin, have been found in OSCC tissues, indicating the crucial role of angiogenesis in tumor growth and progression [13,14].

MVD is a quantitative measure that characterizes the extent of angiogenesis within neoplastic tissue [15]. Over the past decade, a plethora of studies have demonstrated that MVD may serve as a reliable, independent prognostic factor for overall (OS) and disease-free survival (DFS) in primary tumors. These investigations have revealed a significant association between elevated intratumoral microvascularization, the presence of metastases, and unfavorable prognosis in breast cancer and various other forms of solid tumors [16,17,18,19]. However, some studies have suggested a potential correlation between elevated vascularity and improved prognosis in solid tumors [20,21]. Elucidating tumor heterogeneity and spatial architecture of neoplastic tissue is a paramount concern that has gained considerable scientific attention in the last decade. A detailed understanding of the spatial organization may provide significant implications for the optimization of cancer therapy [22,23]. In this context, angiogenic heterogeneity is a critical component, particularly as vascular patterns dictate the distribution of chemotherapeutic agents within tumor tissue [24]. 

Therefore, the role of tumor vascularization needs to be better understood to identify possible therapeutic strategies and ultimately improve patient outcomes. The aim of the present study was to characterize the vessel quantity and distribution within the tumor center (TC) and the invasion front (IF) of 71 patients with OSCC by immunohistochemical techniques. Furthermore, we assessed the prognostic significance of vessel quantity in relation to survival outcomes, taking into account their specific spatial distribution.

## 2. Materials and Methods

We obtained tissue samples from 71 patients diagnosed with OSCC and treated at the University Hospital of Regensburg, Germany. Ethical approval was granted by the Ethics Committee of the University of Regensburg (reference number: 12-101-0070). 

### 2.1. Histopathological Analysis

The tumor was identified using histological examination from routine pathology diagnostics using hematoxylin-eosin staining. For our study, a representative slide that contained both the tumor center and the invasion front in a well-distinguishable manner was selected.

**Figure 1:**
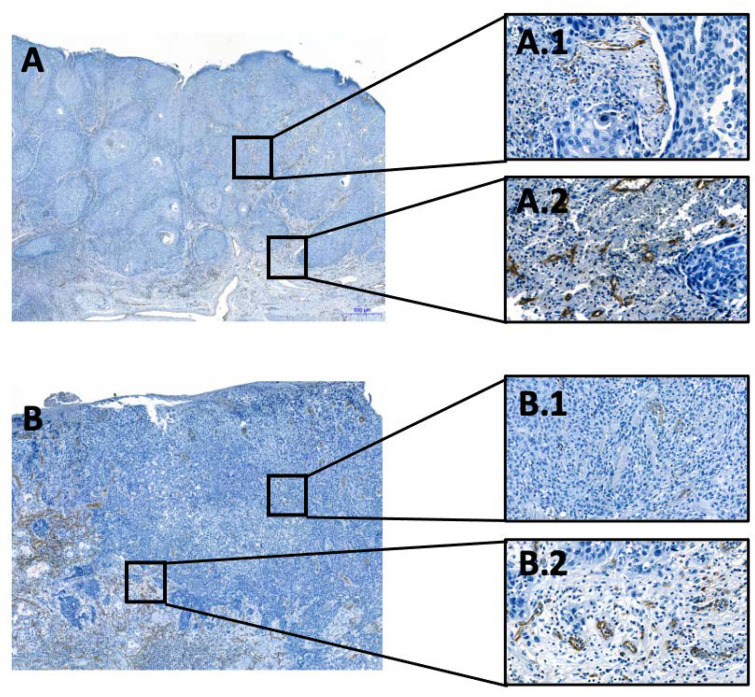
Examples of the evaluated OSCC cases. Visualization of endothelial cells and thus of vessels. (**A**,**B**): Overview of two tissue samples, with the immunohistochemical CD31-reaction at the invasion front, 50× magnification; (**A.1**,**B.1**): Overview of two tissue samples, with the immunohistochemical CD31-reaction in the tumor center, 200× magnification. (**A.2**,**B.2**): Overview of two tissue samples, with the immunohistochemical CD31-reaction at the invasion front, 200× magnification. All immunohistochemical reactions mark positive cells in brown.

To assess the number of vessels in the tissue, we utilized the CD31 antibody (Dako Monoclonal Mouse Anti-Human CD31, Clone JC70) to identify endothelial cells of the vessels. All immunohistochemical staining was carried out on 2–4 µm-thick tissue sections from formalin-fixed (4% neutral buffered formalin) paraffin-embedded tissue blocks. This procedure is a part of our established routine diagnostics at the institute, as reported previously [25]. The staining was performed using the Roche Ventana Benchmark Ultra automated slide stainer (Ventana Medical Systems, Roche, France) with the OptiView DAB IHC Detection Kit (Roche, Meylan, France). The specimens were first deparaffinized, rehydrated, and then underwent antigen retrieval by heat treatment for 32 min with Tris-EDTA Borate Buffer (pH 8–8.5). After incubation with the primary antibody, the nuclei were counterstained with hematoxylin (as shown in Figure 1).

The resulting slides were scanned using the 3DHISTECH Ltd. Pannoramic slide scanner 250 and analyzed using virtual microscopy software (3DHISTECH Ltd. Case Viewer Ver.2.2, Budapest, Hungary), as demonstrated in Figure 1. To determine the number of vessels, two pathologists (A.M. and C.B.) independently identified twenty high power fields (HPFs)—10 from TC and 10 from the IF. The IF and TC were distinguished, and the vessels in each HPF were counted manually. The presence of perineural, vascular, and lymphatic infiltration was assessed during the analyses and then compared to the patient’s pathology report. The investigators were blinded to patients’ outcomes.

### 2.2. Statistical Analysis

To perform statistical analysis, we recorded and analyzed all obtained results using SPSS (IBM Statistics version 29.0), GraphPad Prism (GraphPad Software, San Diego, CA, USA), and STATA (StataCorp, 2015, release 14). Categorial variables are presented as number (n) and percentage (%), while continuous variables are presented as mean and standard deviation (±SD). We tested for the normal distribution of continuous variables using the Shapiro–Wilk test. The t-test and Mann–Whitney U-test were performed for parametric and nonparametric values, respectively, to examine differences between vessel distribution in the tumor center and invasion front. We used Spearman’s rank correlation to assess the correlation between vessel count in the TC and IF. Hazard ratios were calculated using the univariable and multivariable Cox regression models. All variables from the univariable analysis with *p*-values ≤ 0.10 were included in the multivariable model. The Kaplan–Meier estimator was used to generate survival curves. The time between primary treatment and death was considered as OS. DFS was defined as the time from curative-intent treatment until the recurrence of disease or death. The mean follow-up duration was 3.9 years (±3.01) for OS and 3.56 years (±3.19) for DFS. We considered *p*-values ≤ 0.05 as statistically significant.

## 3. Results

### 3.1. Study Population

Details of the patient characteristics are provided in Table 1. The mean age of included patients was 67 years (±9.55), ranging from 47 to 91 years at the time of diagnosis. Out of the 71 carcinomas analyzed, three (4.2%) were considered well-differentiated (G1), 58 (81.7%) were moderately differentiated (G2), and ten (14.1%) were poorly or undifferentiated (G3). Using the tumor size and TNM-Classification system according to the UICC 7th Edition [26], 23 (32.4%) carcinomas were categorized as T1, 22 (31.0%) as T2, nine (12.7%) as T3, and 17 (23.9%) as T4a. The UICC staging resulted in 16 (22.5%) patients in Stage I, 11 (15.5%) patients were in Stage II, 13 (18.3%) patients were in Stage III, and 31 (43.7%) patients were in Stage IV.

### 3.2. Spatial Heterogeneity

First, we investigated the difference in MVD between the TC and the IF. The mean value of vessels per HPF was 2.9 (±2.1) and 10.3 (±5.1) for the TC and IF, respectively. All samples showed significantly more vessels per HPF in the IF than in the TC (*p* < 0.001). Furthermore, more vessels in the TC correlated with more vessels in the IF (Rho: 0.789; *p* < 0.001) (Table 2). 

### 3.3. Prognosis

Next, we evaluated the prognostic relevance of MVD on OS and DFS. Therefore, we dichotomized MVD in TC and IF along the mean value as previously described [25]. All patient characteristics of the dichotomized groups are shown in Supplementary Table S1.

**Figure 2:**
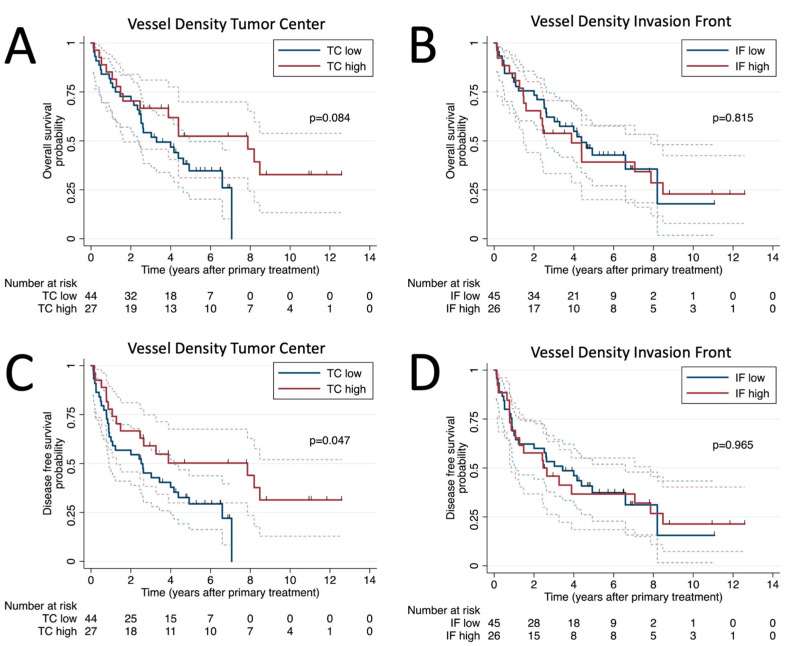
Survival analysis. Kaplan–Meier plots of overall survival for vessel density at tumor center (**A**) and invasion front (**B**) and disease-free survival probability for vessel density at tumor center (**C**) and invasion front (**D**).

The univariate Cox regression analysis of patient demographics and histological characteristics is shown in Table 3. Among all assessed variables, nodal stage and high MVD in the TC were found to be significant predictors of OS, while only high MVD in the TC was a significant predictor of DFS. Specifically, patients with N3 showed worse OS (hazard ratio [HR]: 2.95; 95% confidence interval [CI]: 1.41–6.17, *p* = 0.004), while those with high MVD in the TC showed a better DFS (HR: 0.51; 95% CI: 0.27–0.99, *p* = 0.047) (Figure 2C). Additionally, patients with blood vessel invasion showed a non-significant trend of worse OS (HR: 3.33; 95% CI: 0.99–11.22; *p* = 0.053) and DFS (HR: 2.57; 95% CI: 0.78–8.47; *p* = 0.121). 

Next, we performed a multivariable analysis by including all variables with *p* < 0.100 of our univariable analysis in our multivariable model for OS and DFS, respectively (Table 4). Multivariable Cox regression analysis for OS revealed N3 Stage (HR: 2.82; 95% CI: 1.26–6.31; *p* = 0.012) and high vessel count in the TC (HR: 0.45; 95% CI: 0.22–0.95; *p* = 0.036) as independent prognostic factors for OS. In terms of DFS, N3 (HR: 2.83; 95% CI: 1.36–5.91; *p* = 0.005) and high MVD in the TC (HR:0.43; 95% CI: 0.22–0.84; *p* = 0.014) were also found to be independent prognostic factors.

## 4. Discussion

In the present study, we clearly demonstrated a significantly higher MVD at the invasion front in OSCC compared to the TC. Furthermore, patients with a high MVD in the TC showed significantly better outcomes in terms of DFS. According to the recent literature, this is the first study highlighting the prognostic significance of angiogenic tumor heterogeneity in OSCC patients. 

Spatial heterogeneity of cellular and structural components in the tumor microenvironment are subjects of current scientific discussion [25,27,28]. In our study, we observed a significant increase in MVD within the peritumoral areas. However, we did not observe any significant difference in MVD between different tumor stages. Regarding spatial heterogeneity, Kahler et al. observed differences in vessel distribution between primary colorectal tumors and liver metastases using CD34 as an endothelial marker [28]. Their novel angiogenic model proposes that in primary cancer tissue, the blood vessels are predominantly located near the intestinal lumen, forming a characteristic vascular belt zone, whereas, in liver metastases, hyper-vascularized zones emerge near the invasion front [28]. Although these findings are specific to colorectal cancer, they demonstrate that hyper-vascularized zones tend to affect the marginal zone of the tumor. These findings underline our own observation of elevated MVD in the invasion front of OSCC. Correspondingly, Shieh et al. revealed that vascular hotspots were predominantly located at the peripheral margin of OSCC [29]. However, the authors primarily focused on the temporal evolution of microvessels during disease development and progression by comparing physiologic oral mucosa, leukoplakia, and OSCC tissue samples. Interestingly, peritumoral MVD increased as the disease transitioned from normal tissue to the dysplastic state and subsequently to early cancer. This suggests that angiogenesis may be initiated in the early stages of oral carcinogenesis, specifically in the pre-malignant lesion stage. As a result, the study by Shieh et al. clearly demonstrated that peri- and intratumoral MVD vary during tumor progression [29]. In contrast, Shivamallappa et al. suggested that changes in the angiogenic phenotype occur in carcinomas rather than in the pre-cancerous stage. In their immunohistochemical evaluation, the authors found no statistically significant differences between normal mucosa and leukoplakia but significant differences between the non-malignant lesions and OSCC regarding MVD [30]. A possible explanation for these discrepancies in MVD findings might be attributed to various tumor biological factors of the different cohorts. Additionally, variations in staining methodology may also be a contributing factor to these inconsistencies. The equivocal findings regarding the temporal evolution of MVD and the potential impact of the angiogenic architecture emphasize the urgent need for a better understanding of spatial heterogeneity, both on a genetic and a tissue level.

Spatial heterogeneity of MVD might be caused by a variety of factors. First, angiogenic profiles are affected by the complex interplay between cellular and structural players of the tumor microenvironment [31]. Interestingly, from a pathophysiological point of view, our findings align with prior research presenting spatial differences in immune cell distribution [20,32] since previous findings of our group demonstrated increased macrophage infiltration at the invasion front and their polarization towards M2 macrophages. This type of macrophage is known to play an important role in tumor angiogenesis and thus could be the reason for the higher MVD in this area [22,25]. In brief, tumor-promoting M2-like tumor-associated macrophages (TAMs) are recruited by the tumor and activated by factors secreted from the tumor microenvironment. Consequently, they release different angiogenic factors, including VEGF-A, TGF-β, TNF-α, and others, which stimulate the formation of new blood vessels. Furthermore, TAMs secrete metalloproteinases such as MMP9 that play a critical role in tissue remodeling by degrading the ECM, creating space for newly formed blood vessels and metastasizing tumor cells [33,34]. Based on these findings, we assume that a high immune cell count leads to an increased MVD in OSCC, which has been previously described in other cancer entities [35]. However, further research is needed to confirm this observation and to develop new treatment strategies, such as repolarizing macrophages towards an anti-cancerous M1 phenotype [36].

Following the analysis of the spatial heterogeneity of tumor vessels, its impact on prognosis, specifically OS and DFS, was analyzed. Our findings clearly demonstrated that a high intra-tumoral MVD is a positive predictor of DFS. With a view to the recent literature, our study is the first to analyze MVD as a prognostic factor in OSCC in relation to spatial heterogeneity of both tumor center and invasion front.

MVD was first used as a prognostic factor in breast cancer patients by Weidner et al. in 1992. In that study, MVD was significantly associated with overall and relapse-free survival in all patients [15]. Since then, numerous studies on MVD in various oncological disciplines have been conducted [37,38,39]. A recent meta-analysis in HNSCC patients demonstrated that high MVD is associated with poorer 5-year OS and PFS [26]. In contrast, we did not find any statistically significant correlations between OS and MVD, which may be due to several factors. First, the meta-analysis compared different angiogenic factors, such as CD31, CD34, CD105, and F8. Only two of the 14 studies examined OSCC exclusively, using CD34 and CD105 as markers. Furthermore, these investigations did not take into account the spatial distribution of MVD.

Evans et al. examined MVD and lymph vessel density of HNSCC tissue samples in the peritumoral area, which they defined as being less than 500 µm away from the tumor border but not within the tumor [27]. Statistical regression models indicated that high peritumoral MVD was associated with lymph node metastases, while low peritumoral MVD was related to DFS. The authors concluded that peritumoral and intratumoral vessels may have distinct effects on disease progression. Furthermore, they emphasized the importance of including the TC in further investigations to better understand the differences between the TC and tumor periphery, which we addressed in our present study [27]. With respect to our results, it becomes clear that the recommendation made by Evans et al. to consider the intratumoral area was indeed valid. Our findings did not show a significant correlation between peritumoral MVD and DFS. Unexpectedly, a high intratumoral MVD was associated with better DFS in our OSCC cohort. However, most of the literature considers a high MVD as a negative prognostic factor for a variety of cancer entities, including OSCC [12,15,38]. Many highly angiogenic tumors correlate with a good response to AAT, such as VEGF inhibitor bevacizumab [40,41]. Despite its high MVD, OSCC is known to show an inadequate response to AAT [42]. Our results suggest that taking into account the spatial heterogeneity might bring a new perspective to elucidate the interaction of MVD, survival, and AAT response. In a pathophysiologic context, the association between therapy response and MVD has been discussed controversially. Bevacizumab failed as monotherapy, whereas in combination with chemotherapy, it improved patient outcomes [10]. It may seem paradoxical that the effectiveness of chemotherapy could be improved by an agent that destroys the blood vessels responsible for delivering chemotherapeutic drugs to the tumor cells. In this context, the “vascular normalization hypothesis” proposes that abnormal blood vessels, but not the quantity, contribute to tumor growth and metastasis. Targeting these pathological vessels with anti-angiogenic agents to normalize their structure and function can improve blood flow to the tumor and enhance the delivery of cancer treatments [43]. Future investigations should aim to identify and modify the ultrastructure of pathologic vessels. Normalizing their structure may enable the delivery of therapeutic agents at sufficient concentrations to the tumor site. However, several challenges in the context of controlled neovascular remodeling remain to be addressed [44].

There were certain limitations to our study. Firstly, the retrospective study design at a single institution involves inherent biases. Secondly, the sample size of our study was relatively small, which might affect the broad applicability of our results. However, our sample size was similar to other patient cohorts that were evaluated in the existing literature. In the future, larger prospective studies are necessary to confirm our findings. Immunohistochemistry (IHC) remains the method of choice for quantifying MVD due to its simplicity and cost-effectiveness. However, IHC carries some potential drawbacks that must be considered. Firstly, it only examines tumor slices, which may not be entirely representative of the 3D tumor architecture. Secondly, despite numerous discussions, a definitive angiogenesis marker has yet to be identified, leading to some difficulty in result comparison. Moreover, manual identification of hot spot regions and vessel counting by pathologists may introduce subjective bias [12]. Therefore, future research should consider sophisticated methods, such as algorithm-based identification of hotspots and vessel counting, as well as computational 3D reconstruction, to elucidate the spatial distribution of blood vessels within tumors [45,46].

## 5. Conclusions

The present study shows that the spatial distribution of vessel density differs between the tumor center and invasion front of OSCC. While the invasion front shows a high vessel density, the tumor center is sparse in vessels. Based on our findings, MVD could be considered a prognostic factor in OSCC. In this context, we identified a high intratumoral MVD as a positive prognostic factor in DFS. The investigation of genetic expression data is warranted to validate our results and explore how these results can be leveraged to improve patient therapy and outcomes.

## Figures and Tables

**Table 1:** Demographic and clinical characteristics of included patients.

Variables/Categories	Total	
	n	(%)
**Number of patients**	71	(100.0%)
**Age**		
≥70	26	(36.6%)
<70	45	(63.4%)
**Gender**		
male	53	(74.6%)
female	18	(25.4%)
**Tumor spread (T-Stage, UICC 7th Edition)**		
T1	23	(32.4%)
T2	22	(31.0%)
T3	9	(12.7%)
T4a	17	(23.9%)
**Nodal Stage (UICC 7th Edition)**		
N0	39	(54.9%)
N1	12	(16.9%)
N2	6	(8.5%)
N3	14	(19.7%)
**Metastasis Stage (UICC 7th Edition)**		
unknown	10	(14.1%)
no spread	50	(70.4%)
any spread	11	(15.5%)
**Tumor Grade**		
G1	3	(4.2%)
G2	58	(81.7%)
G3	10	(14.1%)
**UICC-Stage (7th Edition)**		
I	16	(22.5%)
II	11	(15.5%)
III	13	(18.3%)
IVA	31	(43.7%)
**Perineural invasion**		
no	66	(93.0%)
yes	5	(7.0%)
**Lymph vessel invasion**		
no	55	(77.5%)
yes	16	(22.5%)
**Blood vessel invasion**		
no	68	(95.8%)
yes	3	(4.2%)

**Table 2:** Mean, maximum, minimum, and standard deviation of evaluated vessels per HPF.

	n	Mean	Min	Max	SD
TC	71	2.9	1.1	12.6	2.1
IF	71	10.3	4	31.1	5.1
		**Wilcoxon singed-rank test**		**Correlation**	
	**n**	***p*-Value**		**Rho**	***p*-Value**
TC vs. IF	71	<0.001		0.782	<0.001

TC: tumor center, IF: invasion front, Min: minimum, Max: maximum, SD: standard deviation.

**Table 3:** Univariate Cox regression of all patient characteristics.

Variables/Levels	n	OS			DFS		
		HR	(95% CI)	*p*-Value	HR	(95% CI)	*p*-Value
**Number of patients**							
**Age**	71						
≥70 vs. <70 (ref)		1.27	(0.68–2.36)	0.454	1.17	(0.65–2.13)	0.596
**Gender**	71						
male vs. female (ref)		2.01	(0.93–4.34)	0.076	1.62	(0.80–3.26)	0.178
**Tumor spread (T-Stage, UICC 7th Edition)**	71						
T2 vs. T1 (ref)		0.86	(0.41–1.82)	0.692	0.73	(0.36–1.49)	0.390
T3 vs. T1 (ref)		0.89	(0.29–2.69)	0.837	0.65	(0.22–1.95)	0.445
T4 vs. T1 (ref)		1.57	(0.73–3.35)	0.246	1.39	(0.67–2.86)	0.377
**Nodal Stage (UICC 7th Edition)**	71						
N1 vs. N0 (ref)		1.48	(0.65–3.36)	0.351	1.20	(0.54–2.69)	0.658
N2 vs. N0 (ref)		1.49	(0.51–4.35)	0.467	1.50	(0.52–4.34)	0.454
N3 vs. N0 (ref)		2.95	(1.41–6.17)	**0.004**	2.78	(1.37–5.63)	**0.005**
**Metastasis Stage (UICC 7th Edition)**	61						
any spread vs. no spread (ref)		1.74	(0.83–3.67)	0.142	1.82	(0.87–3.82)	0.113
**Tumor Grade**	71						
G2 vs. G1 (ref)		1.58	(0.37–6.70)	0.539	1.76	(0.42–7.45)	0.441
G3 vs. G1 (ref)		4.44	(0.91–21.64)	0.065	4.56	(0.96–21.73)	0.057
**UICC-Stage (7th Edition)**	71						
II vs. I (ref)		1.02	(0.37–2.83)	0.964	0.74	(0.28–1.99)	0.554
III vs. I (ref)		0.77	(0.28–2.13)	0.609	0.56	(0.21–1.51)	0.250
IVA vs. I (ref)		1.83	(0.86–3.89)	0.115	1.61	(0.80–3.23)	0.184
**Perineural invasion**	71						
yes vs. no (ref)		0.37	(0.05–2.73)	0.333	0.30	(0.04–2.17)	0.232
**Lymph vessel invasion**	71						
yes vs. no (ref)		0.98	(0.47–2.04)	0.953	1.00	(0.50–2.02)	0.992
**Bloodvessel invasion**	71						
yes vs. no (ref)		3.33	(0.99–11.22)	0.053	2.57	(0.78–8.47)	0.121
**Vessel density TC**	71						
TC high vs. low (ref)		0.54	(0.27–1.08)	0.084	0.51	(0.27–0.99)	**0.047**
**Vessel density IF**	71						
IF high vs. low (ref)		1.08	(0.58–2.01)	0.815	1.01	(0.56–1.84)	0.965

CI: confidence interval, DFS: disease-free survival, HR: hazard ratio, IF: invasion front, OS: overall survival, TC: tumor center.

**Table 4:** Multivariable Cox regression analysis.

Variables/Levels	n	OS			DFS		
		HR	(95% CI)	*p*-Value	HR	(95% CI)	*p*-Value
**Number of patients**							
**Gender**	71						
male vs. female (ref)		1.35	(0.57–3.18)	0.496	-	-	-
**Nodal Stage (UICC 7th Edition)**	71						
N1 vs. N0 (ref)		1.38	(0.59–3.24)	0.462	1.08	(0.48–2.45)	0.846
N2 vs. N0 (ref)		1.62	(0.53–4.99)	0.402	1.72	(0.58–5.10)	0.324
N3 vs. N0 (ref)		2.82	(1.26–6.31)	**0.012**	2.83	(1.36–5.91)	**0.005**
**Tumor Grade**	71						
G2 vs. G1 (ref)		0.82	(0.17–3.83)	0.799	1.09	(0.24–4.83)	0.913
G3 vs. G1 (ref)		2.53	(0.46–13.91)	0.284	3.12	(0.63–15.54)	0.165
**Bloodvessel invasion**	71						
yes vs. no (ref)		1.46	(0.37–5.81)	0.589	-	-	-
**Vessel count TC**	71						
TC high vs. low (ref)		0.45	(0.22–0.95)	**0.036**	0.43	(0.22–0.84)	**0.014**

CI: confidence interval, DFS: disease-free survival, HR: hazard ratio, IF: invasion front, OS: overall survival, TC: tumor center.

## Data Availability

Not applicable.

## Supplementary Materials

The following supporting information can be downloaded at: https://www.mdpi.com/article/10.3390/biomedicines11102724/s1, Table S1: Demographic and clinical characteristics of TC low/high and IC low/high groups.
