# Supplementary material for: Immunohistochemical Assessment of Microvessel Density in OSCC: Spatial Heterogeneity of Angiogenesis and Its Impact on Survival
Source: Biomedicines. 2023 Oct 8;11(10):2724. doi: 10.3390/biomedicines11102724 (PMC10604174; doi:10.3390/biomedicines11102724)
Supplement: Supplementary file 1 [file biomedicines-11-02724-s001.zip › biomedicines-2626374-supplementary.pdf]

Supplementary Table S1. Demographic and clinical characteristics of TC low/high and IC low/high groups

	TC low		TC high			IF low		IF high		
Variables/Categories	n	(%)	n	(%)	p-value	n	(%)	n	(%)	p-value
**Number of patients**	44	(100.0%)	27	(100%)		45	(100.0%)	26	(100.0%)	
**Age**										
≥70	17	(38.6%)	9	(33.3%)		19	(42.2%)	7	(26.9%)	
<70	27	(61.4%)	18	(66.7%)	0.653	26	(57.8%)	19	(73.1%)	0.197
**Gender**										
male	35	(79.5%)	18	(66.7%)		34	(75.6%)	19	(73.1%)	
female	9	(20.5%)	9	(33.3%)	0.226	11	(24.4%)	7	(26.9%)	0.817
**Tumor spread (T-Stage, UICC 7th Edition)**										
T1	14	(31.8%)	9	(33.3%)		15	(33.3%)	8	(30.8%)	
T2	14	(31.8%)	8	(29.6%)		14	(31.1%)	8	(30.8%)	
T3	7	(15.9%)	2	(7.4%)		7	(15.6%)	2	(7.7%)	
T4	9	(20.5%)	8	(29.6%)	0.687	9	(20.0%)	8	(30.8%)	0.705
**Nodal stage (UICC 7th Edition)**										
N0	24	(54.5%)	15	(55.6%)		27	(60.0%)	12	(46.2%)	
N1	7	(15.9%)	5	(18.5%)		6	(13.3%)	6	(23.1%)	
N2	9	(20.5%)	5	(18.5%)		8	(17.8%)	6	(23.1%)	
N3	4	(9.1%)	2	(7.4%)	1.000	4	(8.9%)	2	(7.7%)	0.609
**Metastasis stage (UICC 7th Edition)**										

unknown	29	(65.9%)	21	(77.8%)		28	(62.2%)	22	(84.6%)	
no spread	8	(18.2%)	3	(11.1%)	0.641	8	(17.8%)	3	(11.5%)	0.108
any spread	7	(15.9%)	3	(11.1%)		9	(20.0%)	1	(3.8%)	
**Tumor Grade**										
G1	1	(2.3%)	2	(7.4%)		3	(6.7%)	0	(0.0%)	
G2	37	(84.1%)	21	(77.8%)		37	(82.2%)	21	(80.8%)	
G3	6	(13.6%)	4	(14.8%)	0.686	5	(11.1%)	5	(19.2%)	0.358
**UICC-Stage (7th Edition)**										
I	8	(18.2%)	8	(29.6%)		9	(20.0%)	7	(26.9%)	
II	8	(18.2%)	3	(11.1%)		8	(17.8%)	3	(11.5%)	
III	9	(20.5%)	4	(14.8%)		8	(17.8%)	5	(19.2%)	
IVA	19	(43.2%)	12	(44.4%)	0.655	20	(44.4%)	11	(42.3%)	0.854
**Perineural invasion**										
no	41	(93.2%)	25	(92.6%)		41	(91.1%)	25	(96.2%)	
yes	3	(6.8%)	2	(7.4%)	1.000	4	(8.9%)	1	(3.8%)	0.646
**Lymph vessel invasion**										
no	37	(84.1%)	18	(66.7%)		38	(84.4%)	17	(65.4%)	
yes	7	(15.9%)	9	(33.3%)	0.088	7	(15.6%)	9	(34.6%)	0.064
**Bloodvessel invasion**										
no	43	(97.7%)	25	(92.6%)		45	(100.0%)	23	(88.5%)	
yes	1	(2.3%)	2	(7.4%)	0.553	0	(0.0%)	3	(11.5%)	**0.045**
